# On-Field Deployment and Validation for Wearable Devices

**DOI:** 10.1007/s10439-022-03001-3

**Published:** 2022-08-12

**Authors:** Calvin Kuo, Declan Patton, Tyler Rooks, Gregory Tierney, Andrew McIntosh, Robert Lynall, Amanda Esquivel, Ray Daniel, Thomas Kaminski, Jason Mihalik, Nate Dau, Jillian Urban

**Affiliations:** 1 The University of British Columbia, Vancouver, Canada; 2 Children’s Hospital of Philadelphia, Philadelphia, USA; 3 United States Army Aeromedical Research Laboratory, Fort Rucker, USA; 4 Ulster University, Belfast, UK; 5 McIntosh Consultancy and Research, Sydney, Australia; 6 Monash University Accident Research Centre Monash University, Melbourne, Australia; 7 School of Engineering Edith Cowan University, Perth, Australia; 8 University of Georgia, Athens, USA; 9 University of Michigan, Dearborn, USA; 10 University of Delaware, Newark, USA; 11 University of North Carolina at Chapel Hill, Chapel Hill, USA; 12 Biocore, LLC, Charlottesville, USA; 13 Wake Forest University School of Medicine, 575 Patterson Ave, Suite 530, Winston-Salem, NC 27101 USA

**Keywords:** On field validation, Wearable devices, Concussion, Head acceleration events, Best practices, Time windowing, Video verification, Machine learning

## Abstract

Wearable sensors are an important tool in the study of head acceleration events and head impact injuries in sporting and military activities. Recent advances in sensor technology have improved our understanding of head kinematics during on-field activities; however, proper utilization and interpretation of data from wearable devices requires careful implementation of best practices. The objective of this paper is to summarize minimum requirements and best practices for on-field deployment of wearable devices for the measurement of head acceleration events in vivo to ensure data evaluated are representative of real events and limitations are accurately defined. Best practices covered in this document include the definition of a verified head acceleration event, data windowing, video verification, advanced post-processing techniques, and on-field logistics, as determined through review of the literature and expert opinion. Careful use of best practices, with accurate acknowledgement of limitations, will allow research teams to ensure data evaluated is representative of real events, will improve the robustness of head acceleration event exposure studies, and generally improve the quality and validity of research into head impact injuries.

## Summary Statements

This work was part of the Consensus Head Acceleration Measurement Practices (CHAMP) project. The objective of CHAMP was to develop consensus best practices for the gathering, reporting, and analysis of head acceleration measurement data in sport. Subject matter experts were recruited to draft a series of papers on various aspects of the issue. As described in detail in a companion paper,^3^ each team drafted a paper and several summary statements ahead of the CHAMP Consensus Conference, held on March 24–25, 2022 at the Children’s Hospital of Philadelphia. The summary statements were discussed, revised as necessary, and ultimately approved by more than 80% of the vote at the conference

1. A head acceleration event (HAE) is defined as an event/incident that gives rise to an acceleration response of the head caused by an external short-duration collision force applied directly to the head or indirectly via the body in sport, recreational, military, or other activities of interest. Wearable devices are often both kinematically and field validated for direct HAEs and not indirect HAEs due to the limitation of reproducing indirect HAEs in the lab and identifying indirect HAEs on the field, respectively.
2. Kinematic data must be filtered to remove potential false positive recordings and verify valid HAEs. Data windowing, video verification, and pre- and post-processing techniques aid in data validation. Individual verification of HAEs is challenging, and time consuming but improper data validation may lead to errors in estimation of exposure.
3. Video verification serves as an independent verification of HAEs for a given application (e.g., device development, sport setting) and provides contextual information for HAEs. However, video should not be considered ground truth as the confidence in video verification depends on video quality and a robust labelling process. Guided and blinded video verification of head acceleration events are useful components to device performance in an on-field environment.
4. Advanced processing techniques (e.g., algorithms or hardware solutions) have the potential to offer fast and reliable verification of valid HAEs. However, they are often developed for specific wearable devices in specific applications (e.g., collegiate football) and it is best practice to independently validate processing methods for use in the intended application.
5. Before deploying head acceleration measurement devices in an on-field environment, users should establish data collection and analysis protocols according to the activity, resources, and research questions. Additionally, users should ensure 1) the devices are functional, 2) the batteries are charged, 3) the devices are attached securely to the individual, and 4) the wearable device is time-synchronized with other concurrent data sources (e.g., video, GPS systems).

## Introduction

Head acceleration events (HAEs) in sporting and military activities have been studied among researchers for over 60 years.^1,45,47,57–59^ In the early 2000’s, the first commercially-available device, known as the Head Impact Telemetry (HIT) System, was introduced as a means to monitor head acceleration data simultaneously and continuously from American Football athletes on the field in real-time.^29^ During the last two decades, numerous studies have been published using the HIT System to monitor HAEs in American football.^49^ Recent advances in sensor technology and the expansion of the commercial market have allowed researchers to broaden this scope beyond the sport of football by using sensors embedded in a mouthguard, attached to the skin at the mastoid process, worn on the head *via* headband/skullcap, or attached to a helmet.^52^

Wearable devices are now ubiquitous in studies of HAEs. These devices have provided varying levels of ability to measure the kinematics of HAEs and an athlete’s exposure to HAEs across multiple games, training sessions and, in some cases, seasons. Despite their convenience and potential to advance our understanding of head accelerations, brain injury, and their sequelae, properly utilizing and interpreting data from wearable devices requires diligence. Additionally, as research on HAEs in sports develops, there is a great need for consensus on procedures surrounding validation and verification of HAEs measured by devices.

For accurate measurement of HAEs, wearable devices must be validated for (1) kinematics and (2) detection of events. In-lab validation against a gold standard (e.g., instrumented anthropomorphic test device) must be performed to assess the accuracy of kinematics measured from the underlying measurement sensors (i.e. the accelerometer), but the ability of devices to detect and record possible HAEs is best conducted in an on-field, real-world environment. The objective of this paper is to define minimum requirements and best practices for on-field deployment and validation of wearable devices to ensure data evaluated are representative of real events and limitations are accurately defined. Best practices covered in this document include the definition of a verified event, data windowing, video verification, advanced post-processing techniques, and on-field logistics, as determined through review of the literature and expert consensus; these best practices apply to device-recorded events collected *in vivo*.

Traditionally implementation of wearable devices in an on-field environment occurs following laboratory kinematic validation of the underlying sensors (i.e. the accelerometer). Though this is not strictly required, best practice recommendations presented herein assume the underlying sensors have been validated in the laboratory for head acceleration kinematics measurement and may be applied across a wide range of activities. This is because often wearable devices rely on the kinematic signals to identify the HAEs, implying some level of accuracy of the underlying sensors in their ability to measure or estimate head kinematics. Understanding these assumptions, on-field deployment and methods to validate wearable device data should be selected and utilized according to the intent of the research study. Justification of methods and associated limitations should be clearly defined and acknowledged when disseminating research results.

## Defining a Head Acceleration Event

**Figure 1:**
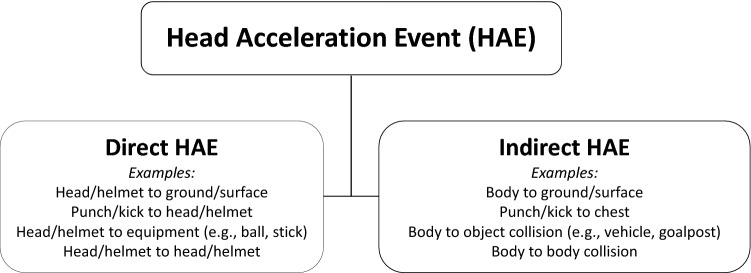
Head acceleration events (HAEs) may be subdivided into direct (i.e., head impacts, involve primary collision with the head or helmet) and indirect (i.e., without head contact, involve primary collision to the individual’s body resulting in inertial motion of the head) HAEs.

A HAE is defined as an event/incident that gives rise to an acceleration response of the head caused by an external short-duration collision force applied directly to the head or indirectly *via* the body in sport, recreational, military, or other activities of interest.^11,14,15,27,30–32,39,72^ For military applications, HAEs considered herein are those that occur in the non-blast environment, though HAEs could be secondary to the blast, such as a direct head impact with a wall following a blast. Direct HAEs, i.e., head impacts, involve primary collision with the head or helmet. Indirect HAEs, i.e., without head contact, involve primary collision to the individual’s body resulting in inertial motion of the head. For example, this may include a fall to the ground or a body-to-body collision. These indirect HAEs are often associated with greater rotational head motions. HAEs can result in a wide array of linear and rotational head acceleration combinations depending on the location and direction of the collision force (Fig. 1).

The definition of HAEs is important for sensor systems as they form the basis for assessing their performance, commonly defined in terms of sensitivity, specificity, or accuracy, and validity. On-field validation of wearable devices is predicated on an assessment of this performance as a measure of their ability to identify HAEs in the field. This fundamentally relies on the verification, though independent means (e.g., *via* in-person notes and/or video), of individual HAEs to quantify this performance. Wearable devices are often both kinematically and field validated for direct HAEs and not indirect HAEs due to the limitation of reproducing indirect HAEs in the lab and identifying indirect HAEs on the field, respectively. However, devices deemed valid from in-lab validation for direct HAEs may reliably measure kinematics of indirect HAEs due to the strict sensor requirements for impact scenarios. Similarly, methods to identify direct HAEs in the field can be applied to indirect HAEs.

## Wearable Devices

**Figure 2:**
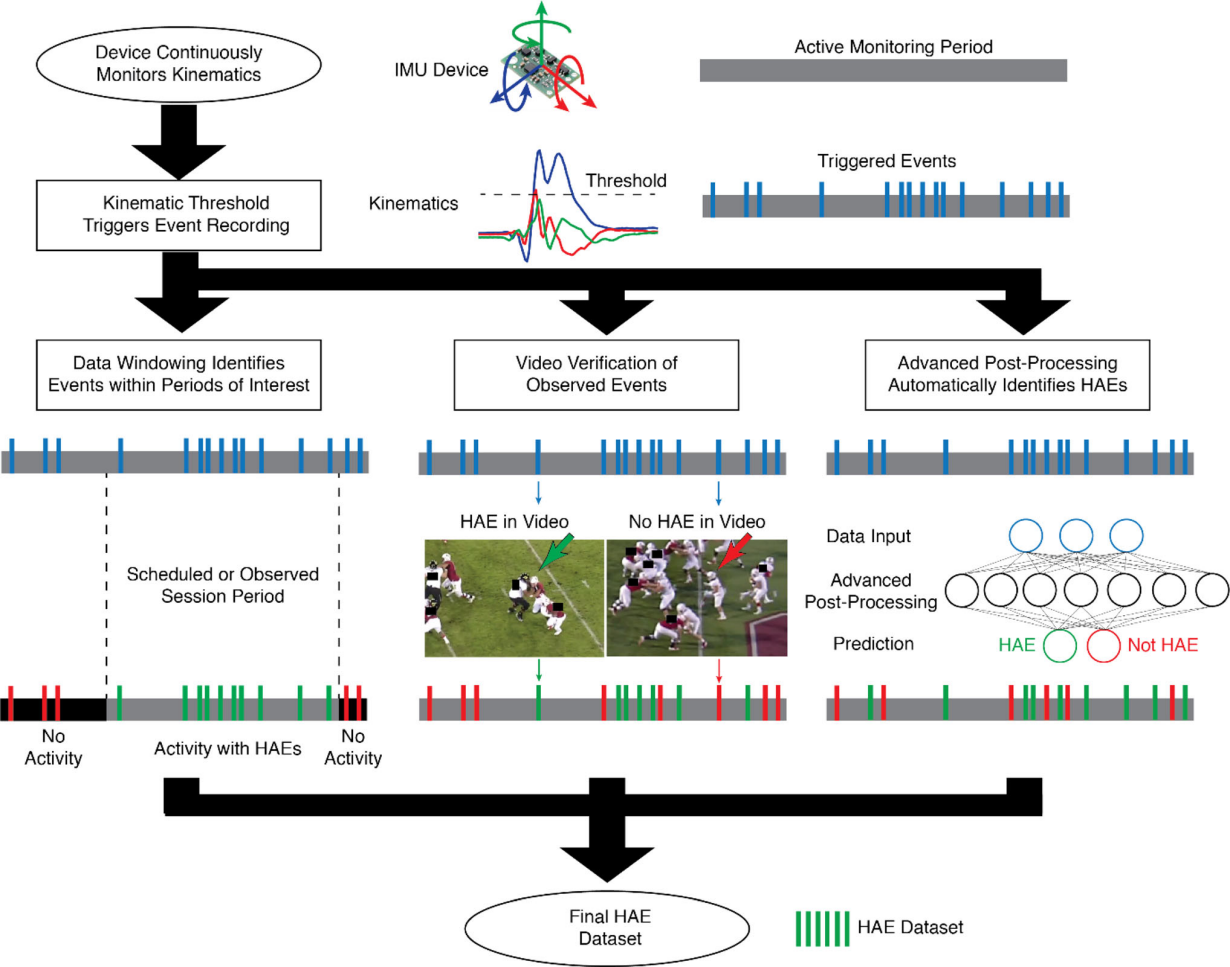
Common methods to establish a final dataset of valid HAE events.

All wearable devices are different; therefore, the device recording, and processing of data for identifying HAEs on the field will vary. Figure 2 depicts the flow of field data for a typical device, which comprises inertial measurement units: linear accelerometers and/or angular rate sensors (ARS). The motion of the device is continuously monitored, and a device-recorded event is recorded to fixed memory and/or transmitted when the device is triggered based on a predetermined threshold. Depending on the device, the recording may be triggered when a single accelerometer channel exceeds a pre-determined linear acceleration threshold^10,19^ or when the transformed data from the array exceeds a pre-determined threshold of resultant linear acceleration.^9,42^ One custom mouthpiece device requires a single accelerometer channel to exceed a linear acceleration threshold for a duration of 3 ms to trigger storing measurements to fixed memory.^61^ The pre-determined recording trigger threshold may be adjustable or set by the manufacturer. A common threshold in the literature is 10g as accelerations under 10g are often associated with indirect HAEs;^27,64^ however, an increasing number of studies have used a 5g threshold as many HAEs are potentially missed, or not recorded, with higher thresholds.^26,44^ Once triggered, the device records some duration of pre- and post-trigger data, which is stored on-board and/or wirelessly transmitted to a sideline receiver or synchronized data collection device.

Few wearable devices allow the raw data recorded at the device location to be accessed directly, but rather perform post-processing of the data either on-board the device^6^ and/or when the data is uploaded to the manufacturer’s server *via* a sideline receiver^25^ or synchronized data collection device.^5,61^ Initially, post-processing typically involves filtering of the time-series data from the individual accelerometer or ARS channels. Devices comprising a linear accelerometer array solve for angular acceleration algebraically while numerical integration is used to calculate angular velocity,^63^ whereas devices comprising ARS directly measure angular velocity and these signals are differentiated to calculate angular acceleration.^10^ Linear acceleration is then transformed from the location of the linear accelerometers to the center of gravity of the head and may again be filtered.^51^

While peak kinematics exceeding recording trigger thresholds are associated with HAEs, these kinematics can also be produced by the removal or application of wearable devices onto the body, or dynamic motion of the body (e.g., running, jumping).^81^ As a result, devices can record a large number of events, with only a fraction associated with HAEs.^11,18,39,54,61^ This necessitates methods to robustly determine which events are associated with HAEs. Here, we introduce the most common methods (Fig. 2), from simple data windowing to video verification to advanced post-processing techniques (machine learning).

## Data Windowing

Data windowing is a relatively simple method for determining which device-recorded events are associated with HAEs. We have defined five steps of data windowing that incrementally reduce the device-recorded events to possible HAEs. Time-stamped device-recorded events are typically downloaded by date either from an online portal, the manufacturer, a sideline collection system, or the device itself. These data often contain device-recorded events for the duration the device was active (e.g., during a practice or game session) on a particular date. This allows the data to be “windowed” to remove events that occurred outside of time windows of interest. To window the data, at minimum study staff should be present during data collection to note time windows of interest. Current best practice is to have video footage of the data collection to facilitate review of, or confirmation of, data windows after the event. It is also best practice to have video synchronized with the clock of the device (i.e., time-synchronized video) to improve alignment between video and device time stamps.

### Temporal Data Windowing

Temporal windowing (i.e., time-windowing) is a method of retaining device-recorded events that occurred during specific time windows and removing events that occur outside of the time windows. The first step of temporal data windowing is to establish the start and end timepoints of a session (Table 1, Step 1). The second step of temporal data windowing involves removing device-recorded events that occurred during scheduled and unscheduled stoppages (Table 1, Step 2). A scheduled stoppage is one that is built into the session (e.g., halftime, water break), whereas an unscheduled stoppage is one that can occur due to circumstances in the session (e.g., timeout, injury) or the environment (e.g., weather). For the sporting context, relying on scheduled session time is not appropriate as actual sessions seldom run to schedule. For example, a study of head impact device data in soccer recorded 9503 device events during scheduled game times compared to the 6796 recorded during verified game times.^54^ To identify unscheduled stoppages, most sports have a sound (i.e., whistle, buzzer, or siren) to indicate when a stoppage has commenced and play has resumed during a game session, which can be used to establish timepoints by study staff or from time-synchronized video. In addition, some sports have game clocks that are paused during stoppages (e.g., basketball).

**Table 1:** Steps of data windowing.

Type of windowing	Step	Window	Requirements	Examples
Temporal	1	Session time	Start and end timepoints of session	Australian football: remove device events that were recorded before the starting whistle and after the final siren Military Training: remove device events recorded before or after the drill/training/exercise of interest (including instruction time)
	2	Stoppage time	Start and end timepoints of scheduled and unscheduled stoppages	Field hockey: remove device events recorded during halftime Basketball: removing device events recorded during timeouts Cricket: remove device events recorded during a rain break Military Training: remove device events recorded during breaks, instruction time, and down time while waiting on remainder of unit to complete drill/exercise of interest.
Temporospatial	3	Group participation time	Timepoints of when a group of interest enter and leave the area of activity or when the individual leaves an area where certain groups must remain	American football: remove device events for the defensive players when the offensive players are on the field Netball: remove device events recorded by a goal-keeper when the goal-shooter on the same team is taking a shot Military Training: remove device events from support units and groups supporting training activity before switching roles.
	4	Individual participation time	Timepoints of when individuals enter or leave the area of activity during the session	Lacrosse and Rugby: remove device events recorded when a player was on the bench or seeking medical attention Ice Hockey: remove device events recorded when a player is in the penalty box Soccer: remove device events recorded after a player is stretchered from the field with a broken leg Military Training: remove device events recorded while Service Member is in line or waiting on next opportunity to perform drill
	5	Individual active participation time	Start and end timepoints of individuals ‘actively’ participating in the session	Boxing: remove device events recorded when a boxer is in a neutral corner after knocking down the opponent Military Training: remove device events occurring when Service Member is not actively performing or engaging in the drill/exercise of interest.

### Temporospatial Data Windowing

The quality of windowing possible HAE data improves when using temporospatial information to further reduce the number of false events. The third step of data windowing involves establishing time windows in which certain groups (e.g., player positions) are active (Table 1, Step 3). The most common example of this occurs in American football. When following a single team, only one of the three positional groups (i.e., offensive, defensive, and special teams) is on the field per play. Players at younger levels of play may compete across multiple position groups in a single session and/or across the season, so it may be challenging to identify/track groups which may overlap and vary over the data collection period. Conversely, some sports have rules limiting the playing area in which a position may occupy (e.g., lacrosse, netball); therefore, if the play moves to a different area, certain playing positions are no longer active. It is important to note that this may be challenging during practice or training sessions for sports applications as position groups may practice together or at different locations on the field; however, if resources and staffing allow, study staff field notes and time-synchronized video may be used to track activities on-field, including when groups or subsets of individuals take breaks during the data collection period (see Step 2).

The fourth step of data windowing requires obtaining individual participation time, which is when an individual is known to be participating in the activity of interest (Table 1, Step 4). In sporting applications, a log of starting players and subsequent changes (e.g., substitutions, injuries, penalties, and ejections) during games may be collected live by study staff or determined from time-synchronized video. For some sports (e.g., soccer), substitutions are made during breaks in play and can therefore easily be captured by a camera filming the game play. Some sports, however, allow substitutions during live play (e.g., men’s lacrosse, ice hockey), which may present challenges determining accurate timepoints if the active play, or field of view of the video, is remote from the substitution location. In such circumstances, it is recommended practice that additional time-synchronized video of the substitution location is captured. Statistical records are kept for some sports that may note timepoints of interest; however, such records are typically only available for higher levels of play and time-synchronization with device-recorded data maybe challenging. Other technologies can also be used to provide temporospatial information. For example, a previous study using wearable devices in skiers used global positioning system (GPS) data to remove device events that were not recorded on ski slopes.^22^ Conceptually, this method could be applied to field activities as some use GPS trackers,^20^ the data from which could be used to remove device-recorded events that were not spatially associated with the activity of interest. This tool may be challenging for applications requiring indoor data collection.

The fifth step of data windowing is to establish active participation time for an individual (Table 1, Step 5). Although an individual may be present for the activity of interest, they may be remote from the activities susceptible to HAEs and/or not actively participating in the activity. This is similar to temporospatial windowing by group (Step 3), but for individuals. For some applications, the individual participation time and active participation time align (e.g., ice hockey); therefore, this step will have already been achieved if the device-recorded data has been windowed for individual play time.

The best practice for identifying relevant timepoints for data windowing is *via* time-synchronized video collected during the data collection. In-person monitoring of relevant timepoints (e.g., start and end of session, stoppage times) may be completed, and is the minimum requirement to complete data windowing;^4,7,17,40^ however, in-person notes often lack the preciseness needed for verification and full reliance on this method, in the absence of time-synchronized video collected during a session, may introduce error in recorded timepoints and eliminates opportunities for further verification. It also limits the metadata (e.g., contact characteristics; presence of head contact) that may be collected about the session. Additionally, in-person monitoring of events becomes increasingly more challenging with a greater number of individuals instrumented on-field and the size of the field of play/training arena. The usefulness of in-person monitoring may also depend on the activity, level of play, and resources available (including trained personnel). For example, in-person monitoring of header events in soccer may be conducted more easily than in-person monitoring of head impacts in ice hockey or football due to the level of contact and the speed and complexity of action that occurs during a session.

## Video Verification

Video verification of HAEs serves several purposes in the deployment of wearable devices and their on-field validation. As an independent assessment of HAE incidence, video verification is most commonly used to confirm device-recorded events.^16,18,34,43,54,55,61,73^ This form of guided video verification is primarily used to remove false positive events (i.e., an event was recorded by the device but was not an HAE), and carefully examine high severity sensor recordings that are most associated with mild traumatic brain injuries.^56,60^ This can often be used in conjunction with time windowing (which reduces the periods in which video verification is required), but itself requires substantial resources and coordination to deploy additional video recording resources for reliable verification. Outlined best practices in this section are intended to provide the framework for successful video verification.

During video verification of HAEs, contextual information on the HAE can also be described, such as the impact site (e.g., crown of head)^11,18^ and/or the type of HAE (indirect HAE vs. direct HAE, impacting surface).^39,44,54^ Such contextual information can augment device measurements to categorise head acceleration severity in different types of events^44,71,73^ or can be used to develop machine learning classifiers for wearable devices to automatically differentiate events.^28,78^ In some cases, contextual information can also help determine issues with kinematic measurements (e.g., measurements with unusually high frequency content, kinematics indicating a different impact direction) that are usually caused by external factors (e.g., direct impact to a device or decoupling of the device from the head). Despite its many benefits, video verification remains an important but uncommon procedure in the HAE analysis pipeline, because video verification requires significant investment in personnel to video record events and identify events in the videos and some researchers have accepted the validity of devices at face value without thorough evaluation. In their 2020 review, Patton *et al.* estimated that nearly two-thirds of head impact studies did not perform or report observer or video verification of individual device-recorded events.^52^ Still, video verification remains a best practice method for verifying HAEs in order to validate the on-field capabilities of wearable devices and providing contextual data for further HAE analysis.

### Camera Set Up and Time Synchronization

Effective video verification of HAEs depends on a robust pipeline to record and process video footage from sessions. Recording videos involves deploying video camera(s) to the field, but the number, placement, resolution, and frame rate of video recording plays a role in the quality of video footage for future assessment. In terms of number, a minimum of one camera is required to simply collect video of an event, though single camera systems suffer from player occlusions and ambiguities that introduce uncertainty in video verification. Thus, it is best practice to have at least two cameras capturing different views (such as sideline vs. endzone views for American football) such that one camera can account for occlusions or ambiguities in the other.^16,28,39^ Recommended practice is to utilize additional broadcast quality video cameras (4 k resolution, 60 fps) tracking the passage of play where most HAEs occur (e.g., tracking the ball). This increases confidence during video verification, but also drastically increases cost and time for the video verification process. This may also be challenging at lower levels of sports (i.e., youth) and in military environments, where there is limited infrastructure to facilitate multi-angle video collection. Placement of cameras depends on the activity being recorded, but best practice is to tune placements to maximize areas where potential HAEs may occur. It is also recommended practice to place cameras at heights >2 meters to reduce occlusions. As video cameras often record video at a distance, the resolution of video capture plays a critical role in the quality of video for verification. Most prior work utilizes 720 pixel–1080 pixel resolutions for sports such as American football and soccer.^28,39,48,54^ Greater video frame rates also provide more refined identification of the moment of impact, though as most wearable devices record at least 50ms around an impact, minimum frame rates of 30 frames per second (33 ms between frames) are acceptable.

During deployment of video systems, it is critical that video cameras, on-site observer notes, and other devices (primarily wearable devices) are time-synchronized. Current best practice is to reference all video footage and wearable device clocks (HH:MM:SS) from a single machine at some point in time for each data collection (most often before the data collection). However, researchers should be aware that there is often variance between individual device clocks (video, wearables, computers, *etc*.). Thus, it is recommended practice that all video, on-site reference clocks, and wearable device clocks are referenced twice, once before and once after an activity, to account for differential video and wearable device clock drifts. Such time synchronization is important to accurately capture the likely discrete event that triggers wearable devices, as often there can be multiple HAEs in quick succession (multiple direct HAE, or an indirect HAE followed by a direct HAE). Clock referencing can be achieved by having a machine set the internal clock for video cameras and wearable devices, by video recording the machine clock,^34^ or by video recording intentional impacts applied to wearable devices.^16,18,39,48^ It is a best practice to regularly synchronize the machine clock for the wearable device with a global source (e.g., atomic clock).

### Video Verification Process

Once collected, video verification is a laborious process that requires robust training protocols and consistent instructions on how individuals (raters) should verify or confirm HAEs. As video verification is a subjective process, it is best practice that instructions for rating HAEs be made explicit, for example clear instructions for identifying a direct HAE and how to label HAEs when the head is occluded by another object. At minimum, these rating instructions should be reported in literature so that they can be used in other on-field deployments for consistent ratings across studies. For on-field verification, at minimum one rater should video verify each timestamp of each device-recorded event captured by a wearable device that will be utilized in further analysis. These may not necessarily include all device-recorded events, such as in analysis of severe impacts where only high acceleration events are characterized.^12^ It is best practice to have two raters review each HAE to ensure robust coding and agreement. It is further recommended practice to have raters who have some personal or professional experience with the activity to conduct video coding; however, proper training in reviewing and coding video is of utmost importance.^70,74^ Most importantly, having multiple raters can identify those HAEs that are potentially ambiguous, which may lead to inconsistencies in HAE coding. Inconsistencies can often be resolved through a majority decision (if coded by ≥ 3 raters) discussion amongst the raters that reviewed the coding, or independently coded by an expert rater with more personal or professional experience with the activity. Proper training will likely minimize the number of inconsistencies requiring further review.

To ensure consistent and robust verification of HAEs, it is best practice to conduct intra-rater and/or inter-rater reliability analysis prior to video verification, depending on whether single or multiple raters are used respectively. Intra-rater reliability can be assessed for single raters by having a single rater review a video, wait for a period of time, and review the video once more. To assess inter-rater reliabilities, all video raters involved with video verification should review a common video to identify HAEs. The inter-rater agreement can then be used to ensure robustness of video verification procedures, as well as eliminate raters who do not have sufficient agreement with others.^68^ Low agreement within a single rater indicates high uncertainty or ambiguity in either the video or instructions.

### Independent Blinded Video Verification

Thus far, video verification has been discussed in the context of verifying whether device-measured events are associated with HAEs, a process termed guided video verification. Guided video verification is important for identifying false positive HAE events, a component in validating on-field performance of wearable devices (Table 2). However, guided video verification does not quantify false negative events (i.e., HAE occurred but was not recorded by the device) for wearable devices. Blinded video verification wherein entire videos are analyzed independently of the wearable device measurements can be used to identify false negative events.^11,13,28,39,53^ False negative events can occur due to linear acceleration magnitudes lower than pre-defined recording trigger thresholds^13,36,77^ or if events are misidentified by device algorithms and subsequently erased or not provided to the user.

**Table 2:** Truth table categories resulting from guided and blinded video verification methods.

	Video		Video verification method
	HAE observed	HAE not observed	
Sensor			
Recorded	True positive events	False positive events	Guided video verification
Not recorded	False negative events	True negative events	Blinded video verification

While blinded video verification provides additional an assessment of false negatives for a more complete on-field validation of wearable devices, it also requires more resources. For blinded video verification, at minimum each full video should be reviewed by a single rater to determine independently when HAEs occur. However, this approach is susceptible to individual rater biases (as blinded verification will likely use multiple raters due to the sheer volume of video), and thus recommended practice involves the utilization of a 2-stage video verification process.^28,39^ In the first stage, videos are reviewed by single raters with instructions to identify any possible instance of a HAE (high sensitivity) but are blinded to the head acceleration data. This effectively provides a collection of possible HAEs mirroring what wearable devices provide and thus a guided video verification can be subsequently performed in the second stage to confirm first-stage video-identified possible HAEs (high specificity). In both guided and blinded video verification, when multiple video angles are available, it is best practice to review videos together to determine whether an HAE occurred. Recommended practice is to first review videos independently, potentially by different raters, before they are assessed together. This primarily increases the confidence for HAEs that are independently identified in different video views.

One challenge that may arise when classifying HAEs during blinded video verification, is the ability to discern the amount of contact necessary to induce a wearable device recording of a HAE.^72^ When an event is observed on video but not detected or recorded by the device, it is often referred to as a ‘possible false negative’. However, false negatives may be associated with observable and reported concussions. It is not common practice for studies to report the number of possible false negatives, but it is an important aspect of on-field validation to accurately estimate the HAEs experienced by an individual. Prior studies that have reported the number of possible false negatives have simply reported the number of events but have assumed these instances are ‘non-events’ and did not analyze them further.^23,61^ Alternatively, some studies have included these events in the calculation of impact rates or impact frequency, but acknowledge their exclusion from the analysis of head acceleration data.^72^

### Potential Challenges with Video Verification

Collection of video footage for verification of events may be limited by resources and/or the environment being studied. Even when high quality, multi-angle camera views are collected, there are several challenges that must be considered. When reviewing HAEs, it may be challenging to distinguish direct HAEs (i.e., head impacts) and indirect HAEs. Even with high quality video, there will always be uncertainty surrounding the distinction between direct and indirect HAEs. Many prior studies have excluded device recordings from analysis due to uncertainty of the event, or if the event was not clearly visible in film and only evaluated impacts that could be clearly verified with video; studies have reported a range of 6-75% of events recorded by devices that were ultimately included in analyses.^15,18,21,30,41,50,55,67,76^ Reasons for exclusion include insufficient film quality,^2,74^ the event occurred outside of the field of view,^2,31,54^or the characteristic of interest (e.g., head contact) was not clearly visible.^23^ In the event that a device recording was excluded because it occurred outside the field of view, there is a potential for underestimation of exposure, therefore, the methods, assumptions, and limitations need to be clearly described and acknowledged to aid in interpretation of the data by others.

Video verifying wearable device-measured HAE events remains an important tool or both the validation and development of wearable head acceleration measurement devices. However, it is important to keep in mind that video verification itself is a subjective process and should not be treated as an absolute ground truth, but rather an independent measurement of head impact and HAE exposure. Thus, great care must be taken to ensure robustness of video verification processes, much in the same way that HAE from wearable devices are expected to be validated. While we only present methods for identifying HAEs in video here, similar approaches can be utilized to ensure robustness of contextual analysis for HAEs, such as the impact site or the type of head acceleration. Indeed, in treating video verification as an independent measure for HAEs, similar techniques can be applied to cross-validate future novel methods for detecting HAEs, such as acoustic and proximity monitoring.

## Advanced Post-processing Techniques

While both guided and blinded video verification are often used to verify which wearable device-recorded events are HAEs, this method is not universally accessible due to both the high time and cost requirements. Recent advances in post-processing techniques, such as machine learning, have allowed for automated classification of HAEs,^21,24,28,46,62,78,81^ but care must be taken with implementation of these techniques with acknowledgment of their limitations. Importantly, many commercial advanced post-processing techniques are proprietary, but their performance can and should be independently validated on the field in the setting of a deployment prior to extensive use. This will provide confidence in their further use to quickly identify HAEs and reduce the reliance on video verification.

The goal of advanced post-processing is to analyze wearable device-recorded events and determine which are associated with real events (i.e., HAEs) and which are associated with false events, or non-HAEs. While a seemingly simple task, it is complicated by the use of different wearable device form factors and applications in different contexts (i.e., different sports/activities and/or different populations). Commonly, methods are tuned to work for a specific wearable device in a specific application. This is because different wearable devices are known to have different performance *in vivo*^80^ and different applications result in distinct head acceleration characteristics (helmeted vs. unhelmeted sports).^79^ Thus, it is best practice to validate post processing techniques for each device in each specific application.

There are commonly two approaches to advanced post-processing. One approach relies on additional hardware or study design considerations that can help determine if a wearable device is being properly applied. For example, instrumented mouthguards can be equipped with a specialized proximity sensor to detect when the mouthguard is being properly worn as an additional confirmation of true head acceleration.^81^ Sometimes these techniques can be used as a pre-process filter to determine whether to record data (e.g. if the mouthguard detects that it is not being worn, then data is simply not collected). More often, this information is collected and integrated into a post-process decision tree to eliminate a substantial number of false positive events; however, even these hardware-based methods are often not sufficient to eliminate all false positive events.^78^ This has led to a second approach relying on software post-processing methods to identify the unique features of HAEs from extraneous measurements.

Software post-processing methods often use machine learning algorithms that can be grouped into two distinct classes: feature-based classifiers and deep learning classifiers. The former relies on engineered features that are extracted from kinematic signals in order to differentiate HAEs.^28,62,78,81^ Here, the support vector machine is commonly used and is often paired with frequency-based (i.e., peak frequency of acceleration signals) or biomechanics-based (i.e. biomechanical feasibility of events) features. Deep learning classifiers are designed to learn features from the data, which allow them to identify complex relationships in the data for classification purposes.^21,24,28,46^ Here, traditional fully-connected neural networks and more complex convolutional neural networks are commonly used but require expertise in selecting appropriate hyper-parameters (e.g., number of layers, number of nodes, *etc*.).

While most wearable devices incorporate some form of post-processing to identify HAEs, commercial devices often use proprietary algorithms.^11,34,43^ Even for research-based devices with peer-reviewed post-processing algorithms, it is still important to understand how these algorithms are developed and evaluated. To evaluate post-processing algorithms, the sensitivity and specificity are most often reported as they describe true positive rate and true negative rate, respectively (with video acting as an independent measure). As a best practice, all post-processing algorithms should report these two metrics as a measure of the algorithm performance.

There remains some nuance to how these metrics are calculated that is important to highlight. This pertains specifically to the definition of the true positive HAE and true negative extraneous event. For data that are collected *in vivo* on the field, labels for true sensor-recorded events are often conducted using a guided video verification. Under this process, only events recorded by wearable devices are provided a true positive or false positive label for validating post-processing algorithms. However, based on previous blinded video verification work, it is known that many wearable devices do not record a substantial number of HAEs, likely because they do not produce accelerations above recording trigger thresholds.^11,36,39,53,55,61^ Sensitivity and specificity metrics often do not account for these missed events (additional possible false negatives). Second, as the video verification process is subjective in nature, rater biases can be captured by software post-processing. Third, similar to this, wearable device measurements themselves during an observed HAE can be corrupted by other sources of error, producing kinematic signals that are not representative of head acceleration which in turn may corrupt post-processing pipelines. As an example, in mouthguards, it is known that the lower jaw (mandible) can bite down on mouthguards during a HAE and produce a characteristic high frequency oscillation measured by the underlying kinematic sensor independent of the head acceleration.^38,72^ These can be removed with device-specific techniques (matching peak frequencies in the kinematic head acceleration signal with known oscillation frequencies induced by the lower jaw) but are predicated on understanding the origins of these erroneous kinematic signals.^82^ Finally, due to the nature of wearable devices and HAEs, datasets are often heavily biased with a greater proportion of false positives events. This must be considered when both reporting and interpreting validation statistics on post-processing algorithms. For example, an advanced post-processing method could report 95% sensitivity and 95% specificity, but if the validation data are unbalanced with 100 true positive HAEs and 1000 false positives (this type of imbalance is common for many wearable devices), then it is expected that the advanced post-processing method could identify 95 true positive events (sensitivity) as well as 50 false positive events (specificity), meaning that one third of the wearable-sensor identified events are in fact false events (i.e., incorrectly labeled by the algorithm).

Advanced post-processing methods are a convenient way to automatically classify device-recorded events into true HAEs and false events and further facilitate data processing steps toward a dataset that is representative of real HAEs. This is particularly true for large multi-institution datasets where processes such as video verification are logistically impractical. Tiered approaches can be utilized for these larger datasets, wherein a subset of the data is set aside to develop and validate novel advanced post-processing techniques with video verification, and subsequently applied to the remainder of the dataset. Currently, advanced post-processing methodologies are specific to the wearable device and the application, but we provide best practices to assess their performance and highlight several nuances and limitations in their design.

## On-Field Deployment Logistics and Best Practices

Finally, the last component to ensure quality on field data is in having proper deployment logistics. Wearable head acceleration measurement devices intersect with many disciplines, including ‘Digital health’ and ergonomics/human factors, and are not limited to biomechanics, sports science, or medicine.^33,37,69,75^ Wearable devices are being deployed to measure characteristics of gait and physical activities across a broad range of health conditions,^35,65,66^ but the assessment of usability is very limited.^33^ Searches using combinations of “usability”, “head impact”, “on-field”, and “concussion” identified few papers that reported on the system usability of head acceleration devices. Tierney *et al.*. (2021) reported their intention to conduct a study of player comfort and wearability with an instrumented mouthguard.^71^ Australian Football players responded positively to a wearability survey of the X-Patch sensor, but the authors noted on-field in-game issues, such as devices failing, detaching and being lost.^43^

There is considerable interest in the use of wearable devices across many sports, from American football to cricket, as well as military applications. Each environment has a set of unique characteristics that must be considered when selecting a wearable device and its suitability for on-field deployment (Table 3). There has been no formal research regarding on-field deployment logistics and best practices. Much of what can be written on these topics are discussion points in research papers, reflected in the research methods, or come from experience.

**Table 3:** Logistical requirements and activity characteristics when collecting head acceleration data in sporting and military environments.

Logistical requirement	Activity characteristic	Comment
Battery life	Competition, practice, or training event period	Many sports have competition durations between 40 and 90 min. In some sports, e.g., cricket, an athlete may be active for many hours. Practice sessions vary in length as well. Military training applications may span many hours, often resulting in distribution of multiple sensors per day if battery life is insufficient
	Size of field	If the device has continuous transmission from device to base station, field size may require higher powered transmitters.
	Number of sessions per charge	Often data collection plans involve a single session in a 24-h period allowing time for battery recharge. However, some data collection structures involve multiple sessions in a 24-h period. Multiple devices per day may be needed if battery life is insufficient
Memory	Number of HAEs	Per person these may range from one every few hours in projectile sports (e.g., baseball) to potentially several per minute, depending on the trigger threshold for sampling an impact and false event recordings. The device should be configured to capture HAEs for the duration of the activity of interest.
Form factor and attachment	Activity considerations (e.g., protective equipment commonly used, communication requirements, duration of wear, etc.)	Helmet, mouthguard, or external cranium are common points of attachment. If able, consider the best attachment point for a given activity. Adding a small mass to a helmet or mouthguard may be more acceptable to the athlete than attaching a device to the head. Communication among athletes in the sport (e.g. soccer) may influence compliance of the participant if the device interferes with speech. Device comfort may influence the compliance if the sensor system is intrusive or uncomfortable. This may be exacerbated for long duration of wear
Data transmission	Number of participants/athletes	If the objective of measuring HAEs in real-time (e.g. monitoring of high-risk events), then ideally data will be transmitted to the sideline and monitored. If the objective is to passively monitor HAEs for evaluation after the session or season or if real-time monitoring is not feasible, then extracting data after a session is reasonable. A large number of study participants typically leads to longer time frames needed to extract data locally. Ideally, data will automatically be wirelessly transmitted to a centrally located storage unit
	Access to participants/athletes	Often strict schedules of participants limit the availability of individuals to be outfitted with sensors. Working around these schedules is often required in studies that require more time with the participant
	Concussion rules	Theoretically, participants could be removed from the session based on biomechanical head acceleration parameters in the absence of concussion signs and symptoms. Concussion rules may develop in tandem with sensor technology. However, at this time, this practice is not recommended given the lack of a specific biomechanical threshold for concussion

Four critical on-field deployment best practices are to ensure that (1) the device is functional, (2) the battery (or device power supply) is charged, (3) the device (e.g., mouthguard, skin patch) or device-mounted equipment (e.g., helmet) is attached securely and properly to the individual wearing the device, prior to data collection, and (4) the cameras are time-synchronized to the device clock or common source (e.g., atomic clock; see Windowing and Video Verification). For device functionality, the user can, at a minimum, verify each device turns on as it should. In many cases, the user can see if the device is connected to the base station in real-time. If this feature is available, the user can verify device connection. If the device transmits data to the base station in real-time, the user may impact the device such that the base station displays the impact to the user to ensure active communication. If the device does not have real-time data transmission, the user can follow manufacturer instructions to ensure the device is active prior to the data collection session. To assess battery life, users can verify battery change *via* the base station before use. If the base station does not display a battery life indicator, the user can ensure the devices were charging for an appropriate amount of time prior to the event of interest. It is important to note that battery life tends to decrease over time and it is recommended practice to periodically run battery life checks of random devices that are in use for multiple seasons. It is best practice for users to keep a daily log of device function on-field to record if/when a device is determined non-functional for the respective session due to battery death or sensor connection failure.

Finally, proper device attachment is critical.^8^ Many devices are available, and each has its own attachment method (e.g., *via* skin, teeth, helmet). Manufacturers often provide instructions to ensure proper attachment, and it is important to follow these instructions prior to each data collections session. For example, to improve coupling of helmet-mounted devices, the user can conduct helmet fittings according to the helmet manufacturer’s guidelines to ensure proper fit. For mouthguard mounted devices, the user can ensure that the device fits the athlete comfortably and does not easily fall off the upper dentition when inserted. It is recommended practice to monitor proper fit periodically during the data collection period, when possible, and throughout the season to assess and address changes in fit or attachment. If this happens, it is recommended practice to have a protocol in place whereby users can remedy the deficiency during a stoppage in play that does not impact normal gameplay or practice.

In addition to the on-field deployment best practices and general considerations for wearable device selection, the following list highlights important issues with on-field deployment logistics and best practices:

- *Number of staff and training* The number of staff required will reflect the size of the team cohort, the ease of attachment, device-to-system connection and initiation protocols, and available pre-game or pre-practice preparation time. Staff will require training and a high degree of technical knowledge may be required, depending on the system. Consistent device deployment is one important aspect of obtaining reliable data, including fit/application of devices prior to deployment. Thus, it is important that training is standardized across all staff and staff follow standard operating procedures. Standard operating procedures should be available to staff at each data collection event for reference.
- *Wearable device matches the sport and intentions of the study* In some cases, research teams may be limited to a single device. But, if the research team has a choice between various devices, one of several factors to be considered is the sporting environment and the purpose of the study. Some devices are limited to helmeted sports and even certain types of helmets while other devices attach directly to the participant’s head. There are pros and cons to each device and each needs to be carefully considered, along with laboratory assessed device performance.
- *Follow the manufacturers’ instructions for system operation and device attachment* With an increasing variability in available wearable devices, users must be familiar with operation and attachment manufacturer instructions for their specific device. Users should be aware that device fit, and adherence could change over the course of a session or season and should consult the manufacturers recommendations to ensure consistency. Proper fit should be regularly monitored during the season to assess and address changes in fit or attachment. Where possible, proper fit should be monitored during the activity itself as well.
- *Device reference checks* Ideally, each device would be checked for functionality automatically during the device-to-system connection process. In addition, a quality assurance process should be considered to check that the device is calibrated and providing accurate and reliable measurements. A quality assurance process might include random sampling of devices for laboratory calibration checks and randomly assigned video verification of device-recorded events during the data collection period. This may help identify devices that may need attention or participant behavior that needs to be corrected (e.g., chewing on mouthguard, throwing helmet, fiddling with device during session). The sampling protocol would reflect the known error rates in the devices.
- *Keep daily log of data collection activities* At a minimum, event start and end times, device functionality, and individual attendance can be recorded in a daily log to track data collection activities and facilitate data review (see “Data Windowing” section). Many research teams report that they window the data sets based on event times because the devices record ‘impacts’ during warm-ups and breaks. The actual HAEs are often reduced substantially once only event times are considered. Beyond event times, users should consider noting other potential instances where device recorded events might be recorded unintentionally. This will vary widely by the device that is used. Some examples might be team water breaks during practices where players remove mouthguards and helmets, long breaks in play such as injury timeouts or intermissions, or periods in a game where the research team visually verifies the device is not attached properly (see “Data Windowing” section).
- *Personal health information (PHI) data protection, data storage and record keeping, confidentiality, privacy, and informed consent are all important considerations* The research team should ensure the study participants, or their legal guardians have provided informed consent and that data are protected and confidential. Study teams should avoid using participant names that could be displayed on a base station where a bystander could observe real-time impacts. Data sharing protocols need to be considered relative to local procedures and approved by the respective institutional review boards. The study team should consider whether data will be shared with individual participants or at a team or league level. If they are shared, it is best practice to ensure the participant understands the limitations of on-field head impact data and present the data in a way that is understandable to the lay person. The research team should also have awareness of device data storage policies (e.g., use of a cloud architecture) and ensure that local institutional review board regulations and policies are followed for storing human subject data.
- *Hygiene* All wearable devices will be exposed to body fluids. Therefore, it is best practice that infection control procedures are implemented for handling and use. This will generally involve gloves, cleaning, and disposal protocols. It is also best practice to clean devices according to manufacturer recommendations on a regular basis.^54^
- *Data review* It is recommended practice for study team members to conduct preliminary data reviews on a regular basis throughout the data collection window. Data review will assist in team and individual participant management but will also identify any systematic issues that require investigation.
